# Bio-Derived Furanic Compounds with Natural Metabolism: New Sustainable Possibilities for Selective Organic Synthesis

**DOI:** 10.3390/ijms24043997

**Published:** 2023-02-16

**Authors:** Leonid V. Romashov, Fedor A. Kucherov, Kirill S. Kozlov, Valentine P. Ananikov

**Affiliations:** N.D. Zelinsky Institute of Organic Chemistry, Russian Academy of Sciences, Leninsky Prospect 47, Moscow 119991, Russia

**Keywords:** biomass, bioderived chemicals, organic synthesis, furan, HMF, natural products, sustainable chemistry

## Abstract

Biomass-derived C6-furanic compounds have become the cornerstone of sustainable technologies. The key feature of this field of chemistry is the involvement of the natural process only in the first step, i.e., the production of biomass by photosynthesis. Biomass-to-HMF (5-hydroxymethylfurfural) conversion and further transformations are carried out externally with the involvement of processes with poor environmental factors (E-factors) and the generation of chemical wastes. Due to widespread interest, the chemical conversion of biomass to furanic platform chemicals and related transformations are thoroughly studied and well-reviewed in the current literature. In contrast, a novel opportunity is based on an alternative approach to consider the synthesis of C6-furanics inside living cells using natural metabolism, as well as further transformations to a variety of functionalized products. In the present article, we review naturally occurring substances containing C6-furanic cores and focus on the diversity of C6-furanic derivatives, occurrence, properties and synthesis. From the practical point of view, organic synthesis involving natural metabolism is advantageous in terms of sustainability (sunlight-driven as the only energy source) and green nature (no eco-persisted chemical wastes).

## 1. Introduction

The appearance of 5-hydroxymethylfurfural (5-HMF) in the list of platform chemicals has boosted the interest in the chemistry of biomass-derived furanic compounds. Currently, the synthesis and application of furanic compounds is an important branch of green and sustainable chemistry. Indeed, a myriad of papers describes the synthesis of 5-HMF and its derivatives as well as the preparation of new materials on their basis. Due to the increasing attention to the topic, the synthesis and transformations of C6-furanics are regularly reviewed [1,2,3,4,5,6,7,8,9,10,11,12,13,14].

**Figure 1:**
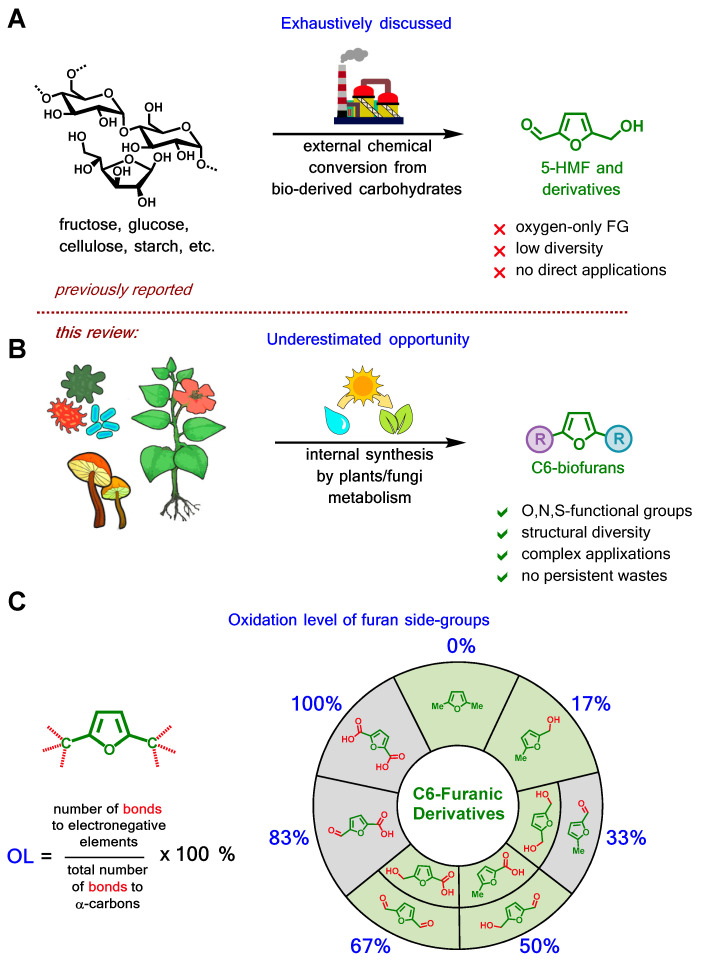
Various approaches to biobased furans: (**A**)—conventional production method of HMF and its derivatives; (**B**)—direct isolation of complex C6-biofurans; (**C**)—classification of furan derivatives via oxidation level metric.

A wide range of naturally occurring carbohydrates serves as a starting point in the chemical conversion of biomass, resulting in bioderived furanics (Figure 1A). Despite all efforts, it remains a typical chemical process with poor E-factor and waste generation. As critically highlighted by Sels and coworkers, the instability of HMF and its derivatives drastically complicates the development of new chemical technologies [15]. Susceptibility of 2,5-functionalized C6-furanic cores to intermolecular condensation leads to contamination, which severely blocks the catalytic centers on the biomass-to-HMF conversion and on the further HMF transformation stages [15,16]. Indeed, any traditional laboratory or industrial synthesis produces waste. Fine chemical synthesis and pharmaceuticals are characterized by poor E-factor (more than 25 kg of waste per 1 kg of the product) [17,18,19,20,21,22]. The processes, including C6-furanic cores, are not exceptions and lead to the formation of a significant amount of waste [23,24,25,26,27,28,29,30,31].

Interestingly, at the same time, 1,4-functionalized C6-furanic moieties can be found among natural compounds themselves. Chemical synthesis by natural metabolism (in plants, fungi, etc.) does not produce persistent wastes (remains are essentially consumed by nature) (Figure 1B). Consideration of natural biosynthetic routes and their incorporation into practice is a great advantage. Finding important and practically relevant natural products followed by their biotechnological production is an attractive methodology for a sustainable future [32,33,34,35]. Therefore, a promising opportunity emerges for the isolation of furanic compounds without external chemical transformations. A considerable number of these compounds demonstrate promising biological activity and could serve as prospective pharmacological substances. The explosive development of furan chemistry in recent decades can change the viewpoint on natural furans. Now, this group of compounds, which were previously discussed as rare and hardly available metabolites, can become attractive from a practical point of view. This review aims to summarize information on natural furanic compounds, which can become targets for total synthesis or biological investigations.

In this focused review, we describe chemically demanding 1,4-functionalized C6-furanic compounds (Figure 1A), which can be found in living nature (plants, fungi, bacteria, marine organisms, etc.) and synthesized by a clean natural method (Figure 1B). In this review, compounds are systematized by the oxidation level (OL) of furan side groups and the type of functional groups present in the molecule (Figure 1C).

## 2. Results and Discussion

The number of oxygen atoms in the C6-furanic derivatives is the key factor for their practical opportunities [36,37,38,39,40,41,42,43,44,45,46]. Depending on the anticipated applications, compounds with lower and higher oxygen contents are of primary interest.

The oxidation level can be calculated as the quotient of dividing the number of bonds with oxygen (i.e., electronegative elements for general use with functionalized derivatives) by the total number of bonds of alpha carbons. The corresponding formula is given in Figure 1C.

For classification purposes, derivatives with the same oxidation level are placed into the same sector (Figure 1C). Compounds whose derivatives have been found in nature are shown on a green background, and the gray background defines compounds that do not occur in nature. The presented scheme provides a general overview of all known nature-occurring furan derivatives. In this review, we discuss nonpolar derivatives followed by compounds with increased oxygen levels.

### 2.1. Oxidation Level of 2,5-Dimethylfuran (OL = 0%)

2,5-Dimethylfuran **1** demonstrates the lowest oxygen content in the C6-furanic series. As a result, this compound is the least polar furan derivative found in nature. Within studies of the potential impact of bioaerosols on health, 2,5-dimethylfuran was found among volatile organic compounds produced by the fungi *Penicillium crustosum* and *Penicillium cyclopium* [47]. 2,5-Dimethylfuran present with the other furan derivatives, and such a mixture could serve as a characteristic indicator for those species. Additionally, **1** is an aroma compound with an odor described as «bouillon» and part of the aroma composition responsible for the aroma of the freshly cut leek *Allium ampeloprasum* (*Alliaceae*) [48]. Freshly cut leek slices stored frozen for one year possess an odor where 2,5-dimethyl furan is one of the most important components of the aroma composition alongside pentanal, decanal and dipropyl disulfide.


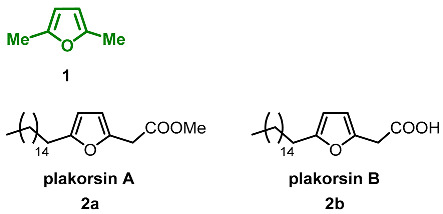


**Scheme 1:**
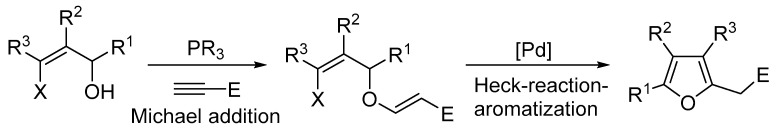
Unified approach to furan natural products via phosphine-palladium catalysis [50].

Furan fatty acids derivatives plakorsins A and B were isolated from the marine sponge *Plakortis simplex* [49]. Synthesis of these natural products was accomplished using elegant tandem of phosphine-catalyzed Michael addition and Pd-catalyzed Heck reaction (Scheme 1) [50]. This unified approach can be applied to the synthesis of tri- and tetra-substituted furans as well.

### 2.2. Oxidation Level of Methyl(Hydroxymethyl)furan (OL = 17%)

This oxidation level is presented by the N-alkylated β-carboline alkaloid **vittacarboline 4**, which was isolated from the ethanolic extract of the fresh flowers of *Hippeastrum vittatum* (*Amaryllidaceae*) [51]. Notably, 3.65 kg of chopped flowers provided 8 mg of pure vittacarboline. Vittacarboline, with its substitution pattern, represents a new structural type within the family since β-carboline alkaloids are not common in the members of Amaryllidaceae.


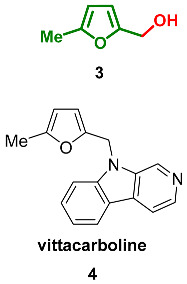


### 2.3. Oxidation Level of 2,5-Bis(Hydroxymethyl)furan (OL = 33%)

2,5-Bis(hydroxymethyl)furan (BHMF) **5** is a symmetric molecule that can be considered a product of the reduction in 5-(hydroxymethyl)furfural (HMF). Since BHMF is a potential monomer for the synthesis of biomass-based polyesters and polyurethanes, its synthesis was described an exceptional number of times [5]. In nature, **5** can be found predominantly in fungi. It was isolated from the fungus *Phellinus linteus*, which grows on mulberry trees [52]; the ascomycete *Xylaria longipes* [53]; the phytopathogen fungus *Colletotrichum acutatum* [54], isolated from strawberry plants; and the marine-derived fungi *Paecilomyces* sp. [55], although the overall bio-production of BHMF is negligible. Extraction of the *Xylaria longipes* culture fluid (18 L) provided 2.9 mg of BHMF. Culture liquid (3 L) extract of Colletotrichum acutatum yielded 7 mg of BHMF. The extraction of 10 L of culture broth of *Paecilomyces* sp. containing both mycelium and supernatant provided a small quantity of BHMF (1.96 mg). Isolated **5** was tested for antimicrobial activity against methicillin-resistant *Staphylococcus aureus* (MRSA), *Escherichia coli*, *Aeromonas hydrophia*, and *Candida albicans* and showed activity only against MRSA with inhibition zone 8 (±0.1) mm.


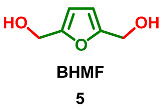


Additionally, **5** is produced by the terrestrial bacteria *Streptomyces* sp. GW 10/2517 [56].

Ethers and acetals

Monomethyl ether of BHMF **6,** as well as BHMF itself, was found in the fungus *Phellinus linteus* [52].


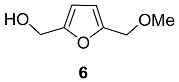


**Scheme 2:**
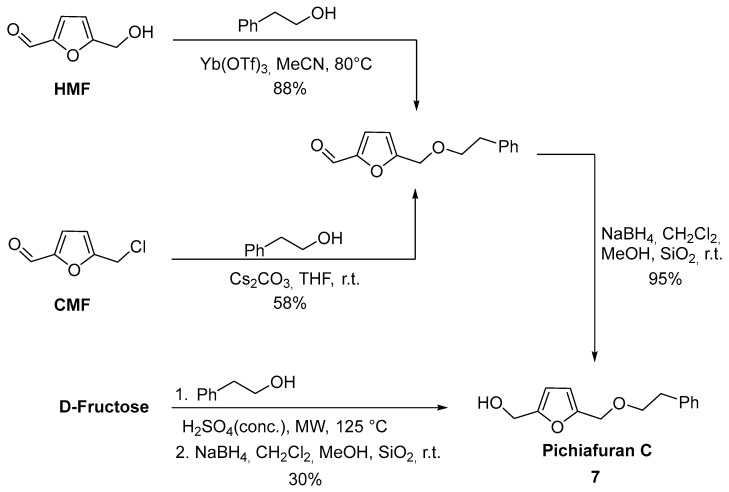
Three ways of the synthesis of Pichiafuran C.

2-Phenylethyl ether of BHMF **7** (Pichiafuran C) is a rare example of monofurano metabolite produced by the yeast *Pichia membranifaciens* derived from a marine sponge *Petrosia* sp. [57]. The C6 monofuran moiety of **7** is likely derived from a triketide. Extraction of the cultivated yeast broth (30 L) provided 5.5 g of crude material, which contained a small amount (0.8 mg) of Pichiafuran C. Compound **7** was investigated for brine shrimp lethality and displayed toxicity with an LD50 higher than 200 μg/mL. Synthesis of pichiafuran C was carried out by Tamariz et al. in three different ways (Scheme 2) [58].


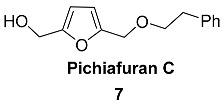


O-β-D-Glucopyranoside of BHMF **8** was isolated from the *Anoectochilus formosanus* (*Orchidaceae*) [59]. The extraction of whole dried plants (2.3 kg) followed by chromatographic purification provided a 59 mg yield of **8**. This furanic glycoside was assayed for inhibition of α-glucosidase and interaction with rat adipocytes; however, no activity was found [59].


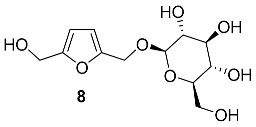


Esters

Mono- and diacetyl derivatives of BHMF **9a** and **9b** are produced by terrestrial gram-positive, aerobic, and nonacid fast bacteria *Streptomyces* sp. GW 11/1695 [60]. Extraction and purification of 25 L of the culture broth produced 24 mg of **9a** and 14 mg of **9b**.


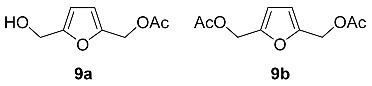


### 2.4. Oxidation Level of 5-(Hydroxymethyl)furfural (OL = 50%)

5-(Hydroxymethyl)furfural (HMF) **10** is formed during the heat-processing of food (baking, smoking, roasting, etc.) Therefore, HMF is often used as an index of heat treatment and deteriorative changes in food such as tomato paste, infant milk, honey, and fruit juices. This compound originates from hexoses via partial dehydration [61]. Therefore, HMF is present in many different food items, including honey, barley, brandy, citrus juices, tomato products, syrup, grape juice, freeze-dried pears, wine, coffee, caramel products, dried fruit, prune juice, and bread. Therefore, everybody is exposed to HMF and some of its derivatives [1,62]. Authentic compound **10** was found in *Schisandra chinensis* (*Schisandraceae*) (whose fruit is known as magnolia berry or five-flavor fruit) [63]. It was found that HMF formed during the processing course. Heating time, temperature and treating solvents all have an effect on the HMF level in the decoction of Schisandra.


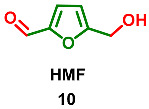


Moreover, HMF **10** was found in red alga *Laurencia undulata*, an edible species used as a folk herb [64]. HMF **10** was thoroughly tested for antioxidant activities and displayed its potential antioxidant character on the molecular, cellular and gene levels.

Ethers

The most simple HMF ether, 5-methoxymethylfurfural **11,** was found in *Achillea millefolium* ssp. *pannonica* (*Asteraceae*), *Cirsium chlorolepis* (*Asteraceae*), *Asparagus cochinchinensis* (*Asparagaceae*) [65] and *Jaborosa Magellanica* (*Solanaceae*) [66]. In 1958, it was reported that furan **11** was present as a component of the roots of *Asparagus lucidus* (*Asparagaceae*), but it was assumed that this product had been formed as an artifact from fructose during the isolation process [67].


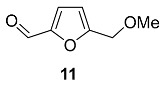


Cirsiumaldehyde **12**, which can be described as a product of the intermolecular dehydration of HMF, was first discovered in the roots of *Cirsium chlorolepis* (*Asteraceae*) [65] and named after it. *Cirsium chlorolepis* roots are used in southwest China folk medicine against various diseases, especially fracture and haematuria. After double extraction and chromatographic separation, the dried roots (5 kg) provided 30 mg of dimeric compound **12**. Later, this compound was found in fruits of *Hippophae rhamnoides* (*Elaeagnaceae*) [68], flowers of *Lonicera bournei* (*Caprifoliaceae*) [69] and tubers of *Gastrodia elata* (*Orchidaceae*) [70].


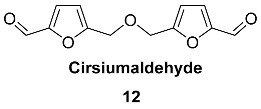


A set of benzylic ethers **13**–**16** were isolated from the rhizome of *Gastrodia elata (Orchidaceae*) [71,72,73]. This rhizome has been traditionally used in Korean and Chinese traditional medicine for the treatment of headaches, migraines, dizziness, epilepsy, and infantile convulsion tetanus [74,75,76] and has many biomedical properties, such as enhancing strength and virility, improving circulation, and facilitating memory consolidation and retrieval [77,78]. Inhibitory effects of **13** for enzymes were tested; however, low activity was found at 100µM concentration with 23% and 39% inhibition ratio for topoisomerases I and II, respectively. Synthesis of compound **13** was performed from HMF and O-protected 4-hydroxybenzylic alcohol using H_2_SO_4_ in CH_2_Cl_2_ [58]. Extraction of 30 kg of dried rhizomes of *G. elata* yielded 4.5 mg of **15** and 4.2 mg of **16**. The presence of a phenolic moiety in these compounds indicates their metabolic relation with lignin. Thioether **16** (sulfur-containing analog of compound **15**) was also found in the rhizome of *Gastrodia elata*. The cytotoxic effects of the isolated compounds **15** and **16** were evaluated in vitro by MTT methods on PC12 cells with no activity found. At the same time, compound **16** concentration dependently protected PC12 cells against H_2_O_2_-induced cell death exhibiting neuroprotective activity [73].


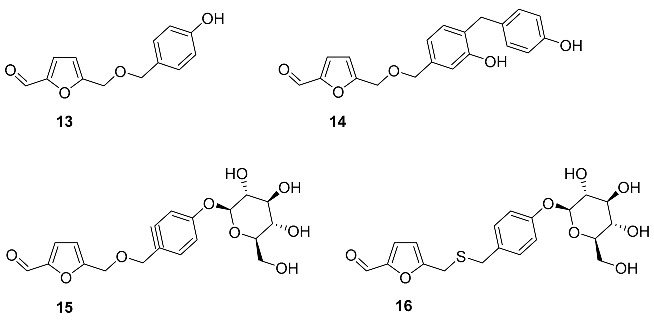


2-Hydroxyphenyl ether of HMF **17** was isolated from the seeds of *Cassia fistula* (*Fabaceae*) [79]. Extraction of 3.5 kg of seeds with methanol followed by chromatography produced 25 mg of **17**. The total synthesis of **17** was performed by reaction of 5-chloro-methylfurfural with catechol and K_2_CO_3_ in acetone solution under reflux.


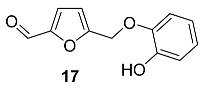


Compound **18** (Acorusin B), which completes the series of aryl-containing HMF ethers, was found in the rhizomes of *Acorus tatarinowii* (*Acoraceae*). Grounded rhizomes (10 kg) extract contained 5.2 mg of Acorusin B. This hybrid norlignan derivative exhibits moderate cytotoxicity against the five cell lines NCI-H1650, HepG2, BGC-823, HCT-116 and MCF-7 [80,81].


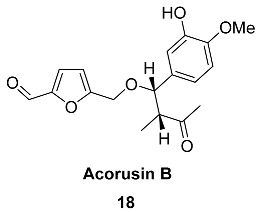


Compound **19** ((±)-Hiziprafuran), which is a furanic derivative of dimethyl malate, was isolated as a racemic mixture from the aerial parts of *Prasium majus* (*Lamiaceae*); the plant has a wide global occurrence and has been used medicinally in Greece as a tranquilizer [82]. The five-solvent extraction of air-dried aerial parts of *Prasium majus* (1200 g) with further purification by column chromatography yielded 70 mg of Hiziprafuran.


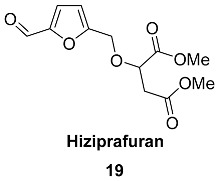


**Scheme 3:**
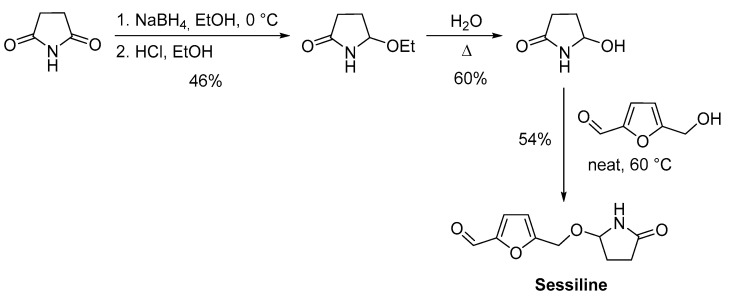
Synthesis of Sessiline **20** from succinimide and HMF.

Compound **20** (Sessiline) is an alkaloid from the fruits of *Acanthopanax sessiliflorus* (*Araliaceae*) [83]. This plant is distributed in Korea, Japan and China and has traditionally been used as a tonic and a sedative, as well as in the treatment of rheumatism and diabetes. Processing of the air-dried powdered fruits (2 kg) provided 23 mg of sessiline. The synthesis of sessiline from HMF and succinimide was described by Ilkei et al. (Scheme 3) [84].


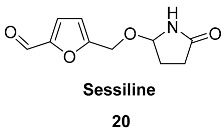


Esters

Compounds **21**–**24** (Duabanganals A-D) are HMF esters with saturated and unsaturated fatty acids that were found in the stem bark of *Duabanga grandiflora* (*Lythraceae*) [85]. It was found that dried and powdered stem bark (1.3 kg) contained Duabanganals A (3 mg), B (4.6 mg), C (2.1 mg) and D (4.8 mg), as well as 3 mg of Latifolinal. Duabanganals B and C were evaluated for cytotoxicity against six cancer cell lines and failed to demonstrate activity.

Stearyl ester **25** (Latifolinal) was isolated from the fruit of Cordia latifolia (Ehretiaceae) [86] and the stem bark of *Duabanga grandiflora* (*Lythraceae*) [85]. Latifolinal can be described as a product of the full hydrogenation of double bonds in Duabanganals A-C. Dried fruits (20 kg) of *C. latifolia* contained 30.5 mg of latifolinal.


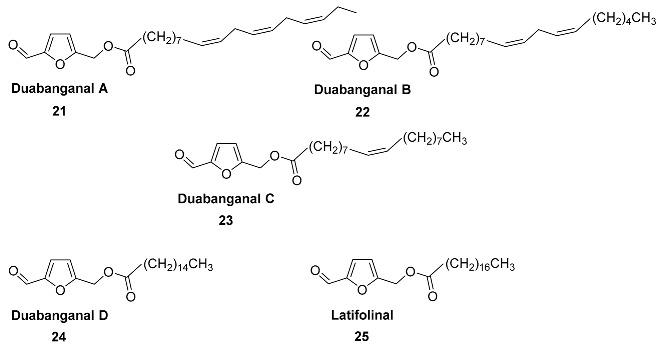


4-Hydroxy-3,5-dimethoxy-(E)-cinnamoyl derivative **26** was found in the seeds of *Raphanus nussatirus* L. (*Brassicaceae*), a traditional Chinese herb that has been used for expectorant, anti-cough and antiasthmatic purposes [87]. The seeds powder of *Raphanus nussatirus* L. (15 kg) extraction produced 60 mg of pure **26**.


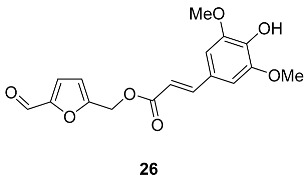


**Scheme 4:**
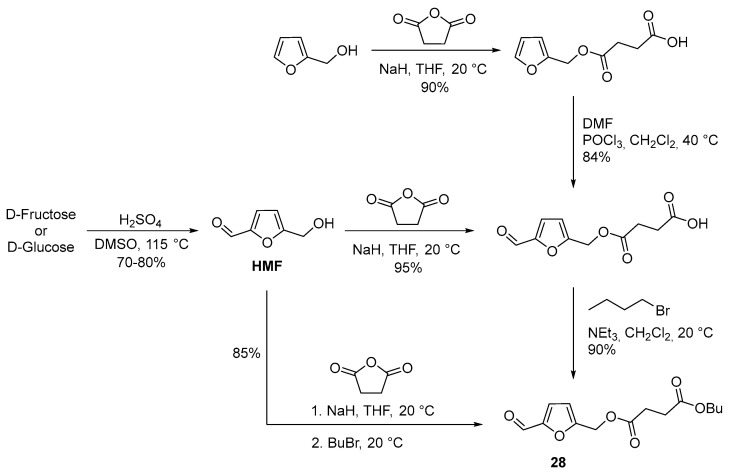
Synthesis of ester **28** from various starting materials.

A series of HMF esters with polycarboxylic acids was found in natural sources. O-(Ethyl succinoyl) ester **27** was isolated from the whole plant of *Ajuga decumbens* (*Lamiaceae*) [88]. O-(Butyl succinoyl) ester **28** was found in the fruit of *Morinda citrifolia* (*Rubiaceae*), also known as noni or great morinda [89]. This fruit has a long history of traditional use in the Hawaiian and Tahitian islands and is thought to exhibit anticancer and immunostimulant activities. The synthesis of ester **28** was performed by Tamariz et al. from various starting materials, including one-pot synthesis from HMF (Scheme 4) [90].


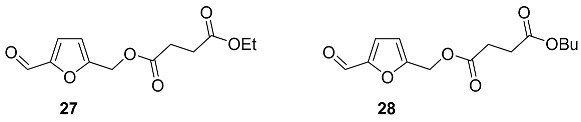


**Scheme 5:**
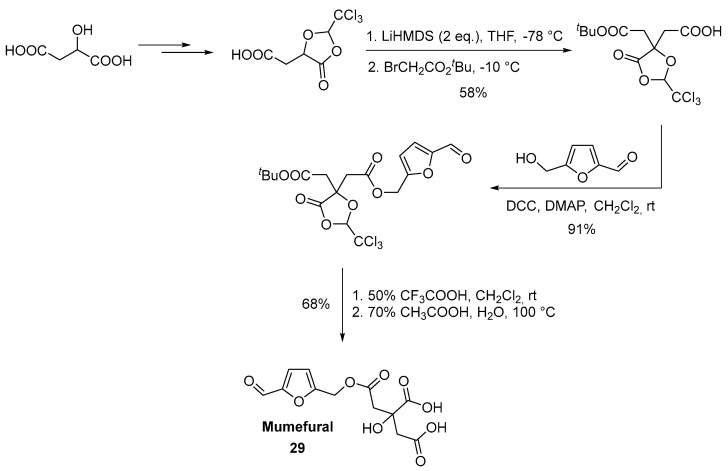
Synthesis of mumefural **29** from malic acid and HMF.

Another polycarboxylic acid whose HMF esters have been found in nature is citric acid. Ester **29** (Mumefural) was isolated from the fruit juice concentrate of *Prunus mume* (*Rosaceae*), Japanese apricot [91]. Mumefural **29** is a sialidase inhibitor and an effective enhancer of blood fluidity [91]. Additionally, ester **29** shows activity against the pandemic influenza A(H1N1) virus [92]. Synthesis of **29** on the basis of malic acid and HMF was performed by Sugimura et al. (Scheme 5) [93]. Diethyl ester of mumefural **30** was isolated from the fruit of *Morus alba* (*Moraceae*) [94].


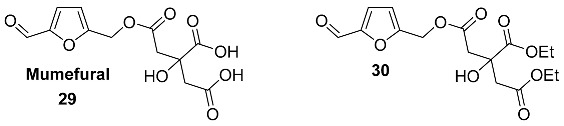


4-Hydroxy-2-methylenebutanoyl derivative **31** was found in the roots of *Polygala tricornis* (*Polygalaceae*) [95]. *Polygala tricornis* is widely distributed in southern China, and its roots are used as a tonic, a sedative, and for preventing dementia in Chinese folk medicines. Isolated compounds were examined for their inhibitory effect on nitric oxide (NO) production induced by lipopolysaccharide (LPS) in BV-2 microglial cells. Compound **31** exhibited pronounced inhibition of NO production with an IC_50_ value of 1.77 μM [95].


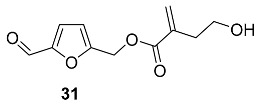


Ester **32** (Asfural) was isolated from an enzyme-treated extract of the fresh bottom parts of *Asparagus officinalis* (*Asparagaceae*) stems [96]. Sucrase C as a cellulase and Macerozyme A as a pectinase were used for the extract treatment. Extraction of 90 kg of fresh asparagus followed by the enzymatic reaction and additional purification yielded 2 mg of Asfural. Asfural **32** was evaluated in terms of HSP70 mRNA expression-enhancing activity in HL-60 cells. Compound **32** significantly increased the expression level in a concentration-dependent manner. The synthesized R-enantiomer of asfural has shown significantly lower activity.


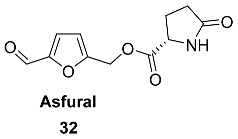


Glycosides

Glycosides of HMF can be described as the products of partial dehydration of the terminal hexose moiety in oligosaccharides. Therefore, this type of HMF derivative is relatively widespread in plants.

A series of D-fructose HMF derivatives O-α-D-fructofuranoside **33**, O-β-D-fructofuranoside **34** and O-β-D-fructopyranoside **35** were isolated from the roots of *Ranunculus ternatus* (*Ranunculaceae*) [97]. The root of this herbaceous plant is used in traditional Chinese medicine as a treatment for lymphatic and pulmonary tuberculosis. Air-dried root powder of *Ranunculus ternatus* contains 4 mg of **33**, 6 mg of **34** and 5 mg of **35**.


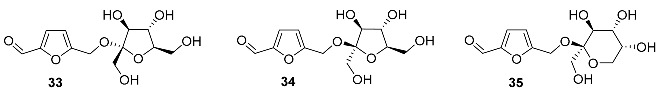


O-α-D-glucopyranoside **36**, O-β-D-glucopyranoside **37**, and O-[α-D-glucopyranosyl-(1→6)-α-D-glucopyranoside] **38** were isolated from *Rehmannia glutinosa* (*Orobanchaceae*), one of the most important traditional Chinese medicines used for multiple therapeutic purposes [98]. Synthesis of HMF glucopyranosides was accomplished via selective dehydration of isomaltulose in DMSO in a strongly acidic ion exchange resin [99,100].


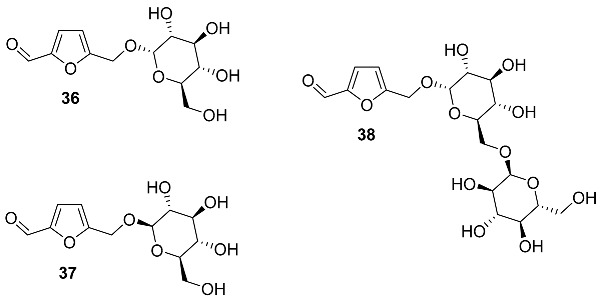


O-[β-D-fructopyranosyl-(2→6)-α-D-glucopyranoside] **39** was found in the fermented beverage from the fruit of *Prunus mume* (*Rosaceae*), a deciduous fruit tree that is distributed widely in East Asian [101]. Traditional raw ingredients are used in herbal medicines in Korea, China, and Japan. A thick beverage was obtained by mixing cleaned fruits of *P. mume* (10 kg) with sugar (7 kg) and storing the mixture in the dark at room temperature for 3 months, followed by residue and seeds filtration from the fermentation broth. After extraction and isolation, 6 mg of compound **39** was obtained.


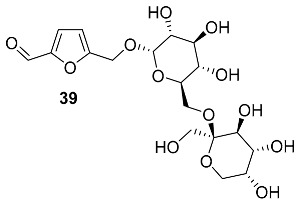


O-(2,6-Dideoxy-3-O-methyl-α-L-lyxo-hexofuranoside) **40** (Calofurfuralside A) and O-(2,6-dideoxy-3-O-methyl-α-L-ribo-hexopyranoside) **41** (Calofurfuralside B) were isolated from the leaves of *Calotropis gigantea* (*Apocynaceae*) [102]. This large shrub is commonly called crown flower or giant milkweed and is used in traditional folk medicines for the treatment of bronchitis, dyspepsia, paralysis, swellings and intermittent fevers. Isolated compounds were tested for cytotoxicity against the PANC-1 human pancreatic cancer cell line, but no significant activity was found.


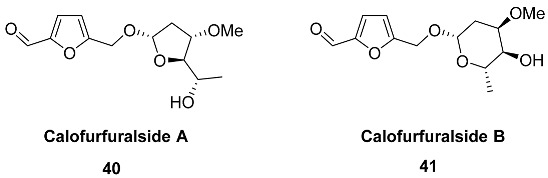


Acetals

Dimeric and trimeric methyl acetals **42** and **43** (Krishnanones A and B) are produced by terrestrial *Streptomyces* sp. GW 21/1313 [60].


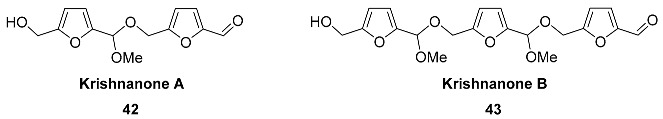


2,3-Butylene acetal **44** (Hypofuran A) was isolated from the marine-derived fungus *Hypocrea koningii* PF04 associated with the sponge *Phakellia fusca*. The biosynthetic precursors of this compound most likely included 5-hydroxymethyl furfural and 2,3-butanediol. Acetal **44** shows modest antibacterial activity against *Staphylococcus aureus* [103]. In addition, Hypofuran A exhibited moderate DPPH radical scavenging capacity with an IC_50_ value of 27.4 μg/mL.


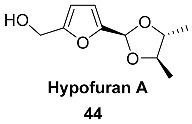


A series of carbohydrate-based acetals and semiacetals **45**–**48** were isolated from plant sources.

Semiacetal **45** and acetal **46** were found in the fruits of *Trichosanthes kirilowii* (*Cucurbitaceae*) [104]. In the ancient East, it was widely used as a folk medicine because its tuber can reduce midterm abortion, and the rind of the fruit can help to cure diseases of the heart and the lung [104]. Exhaustive extraction of the fruits of *T. kirilowii* (20 kg) produced 96.4 mg of **45** and 16.9 mg of **46**.


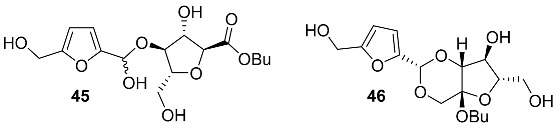


Acetal **47** (Cirsiumoside) was isolated from the roots of *Cirsium chlorolepis* (*Asteraceae*) [65]. This plant is used in southwest China folk medicine against various diseases, especially for fracture and haematuria.


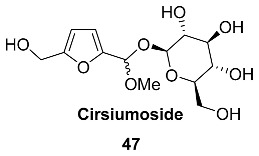


Rare iridoid glucosides **48a**–**d** (Cornusfurosides A–D) were identified in the water extract of the fruits of *Cornus officinalis* (*Cornaceae*) [105]. *Cornus officinalis* fruit, called Corni Fructus (Shanzhuyu) in China, was first recorded in Shen Nong’s Materia Medica approximately 2000 years ago and is often used to tonify the liver and kidney according to the theory of traditional Chinese medicine [106,107]. Glucosides **48a**–**d** were tested for neuroprotective activity, but the results showed that compounds **48a**–**d** exhibited no discernible activities at the concentrations tested [105].


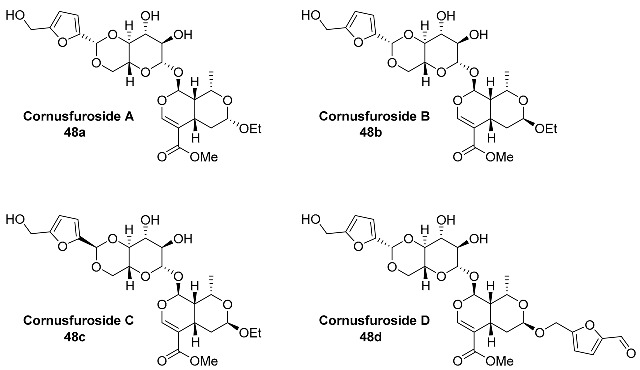


A number of HMF acetals **49**–**52** containing steroid moieties have been found in the roots of several plants.

Acetals **49a,b** (Serfurosterones A and B) are phytoecdysteroids isolated from the methanolic extract of the roots of *Serratula wolffii* (*Asteraceae*) [108]. The phytoecdysteroids, compounds related to insect hormones, are of great interest for their biological activities. The roots of *S. wolffii* (4.7 kg) contain 0.5 mg each of the Serfurosterones A and B; thus, compounds **49a** and **49b** were furnished from the extract with equal yields of 0.00024%.


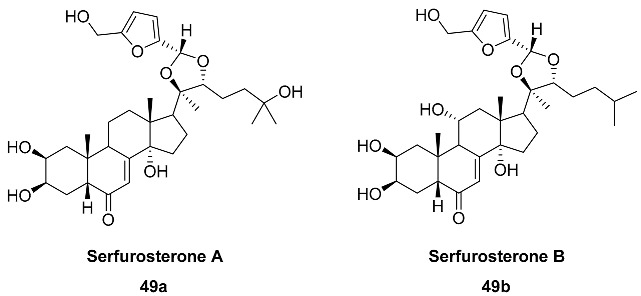


Steroid acetals **50** and **51** (Pulsatilla triterpenic acids A and B) were found in the roots of *Pulsatilla chinensis* (*Ranunculacea*), one of the 50 fundamental herbs used in traditional Chinese medicine [109]. Pulsatillic acids exhibited cytotoxic activities against P-388, Lewis lung carcinoma and human large-cell lung carcinoma [110].


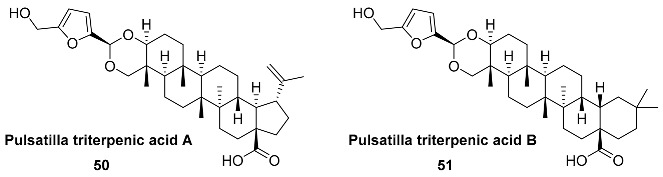


Acetal **52** (Niuxixinsterone D) was isolated from the roots of *Achyranthes bidentate* (*Amaranthaceae*). This herb is used as an important traditional Chinese medicine known as «Niuxi» and has the property of strengthening bones and muscles and ensuring a proper downward flow of blood. Serfurosterone A **49a** was also found in the same source. Niuxixinsterone D and Serfurosterone A were tested for their inhibitory effects against LPS-induced NO production in RAW 264.7 macrophages, and compounds **52** and **49a** exhibited anti-neuroinflammatory activity with inhibited 29.7 and 26.0% NO production, respectively [111].


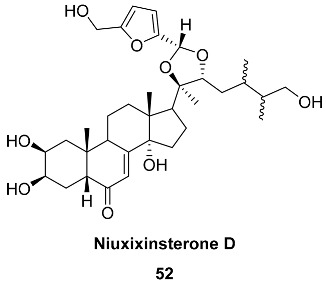


Hydrazones

Rice cultivation of a fish gastrointestinal tract-derived fungus, *Trichoderma* sp. CMB-F563 yielded natural product **53** (Prolinimine A) incorporating a hydrazine moiety [112]. Naturally occurring hydrazines are extremely rare, with only several nonacylated reported examples. The fungal prolinimines represent only the second reported occurrence of a naturally occurring metabolite incorporating an N-amino-Pro residue.


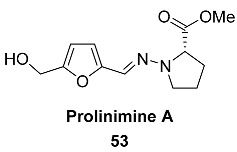


### 2.5. Oxidation Level of 5-Methylfuran-2-carboxylic Acid (OL = 50%)

Methyl 5-methyl-2-furancarboxylate **55** (methyl ester of corresponding acid **54** (MFCA)) is the only known natural compound at this oxidation level. This ester was found among volatile organic compounds released by the bacteria *Saccharopolyspora erythraea* and *S. griseoflavus* [113]. Furan **55** has not been reported as a natural product before.


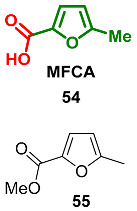


### 2.6. Oxidation Level of 5-(Hydroxymethyl)furan-2-carboxylic Acid (OL = 67%)

5.-(Hydroxymethyl)furan-2-carboxylic acid (HMFCA) **56** (also known as Sumiki’s acid) is produced by the fungi *Aspergillus* spp., *Gibberella fujikuroi*, *Helminthosporium maydis* and *Pyricularia grisea* [114]. Additionally, acid **56** was found in the marine-derived fungi *Epicoccum* sp. [115], *Wardomyces anomalus* [116] and *Cladosporium herbarum* (isolated from the marine sponge *Callyspongia aerizusa*) [117]. HMFCA **56** was found in human urine [118,119]. The analysis showed that Sumiki’s acid is a naturally occurring human metabolite, with a normal excretion range from about 1 to 25 mg/24h. It was proposed that **56** is involved in uronic acid metabolism. Acid **56** shows antitumor activity [114] and antimicrobial activity (against *Bacillus subtilis* and *Staphylococcus aureus*) [117].


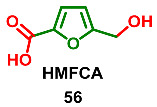


Methyl ester **57** is a metabolite of the phytopathogenic fungi *Curvularia lunata* [120] and *Hericium erinaceum* [121]. This furanic derivative acts as phytotoxin, causing chlorophyll degradation in the maize leaves. Convenient synthesis of ester **57** via oxidative esterification of HMF was recently published [122].


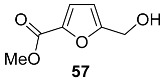


Acetyl derivative **58** (acetyl Sumiki’s acid) was isolated from the marine-derived fungi *Cladosporium herbarum* (isolated from the marine sponge *Callyspongia aerizusa*) [117] and *Epicoccum* sp. [115]. In addition to HMFCA itself, compound **58** is antimicrobial (against *Bacillus subtilis* and *Staphylococcus aureus*) [117].


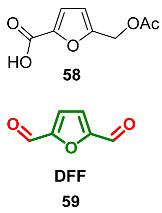


### 2.7. Oxidation Level of 2,5-Diformylfuran (OL = 67%)

DFF double hydrazine **60** (Prolinimine B), as well as Prolinimine A **53,** were isolated from the fish gastrointestinal tract-derived fungus *Trichoderma* sp. CMB-F563 [112]. Such furans are exceptionally rare among natural products, so this compound is the only example of a natural C6-furanic compound at this oxidation level.


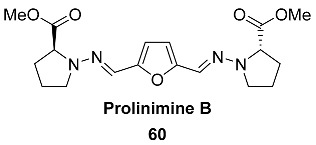


### 2.8. Oxidation Levels of 5-Formylfuran-2-carboxylic Acid and Furan-2,5-dicarboxylic Acid (OL = 83% and 100%)

5-Formylfuran-2-carboxylic acid **61** (FFCA) and furan-2,5-dicarboxylic acid **62** (FDCA) are the most oxidized C6-furanic derivatives. To the best of our knowledge, there are no natural C6-furanic derivatives at these oxidation levels. Since biofurans, in many cases, are the products of partial dehydration of hexoses and their derivatives, it could be assumed that the absence of FDCA and related compounds are caused by the lack of corresponding aldaric acids in natural sources. The absence of FFCA derivatives is more challenging to explain because the corresponding uronic acids, including fructuronic acid, are well known.


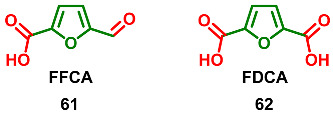


## 3. Conclusions and Outlook

**Figure 2:**
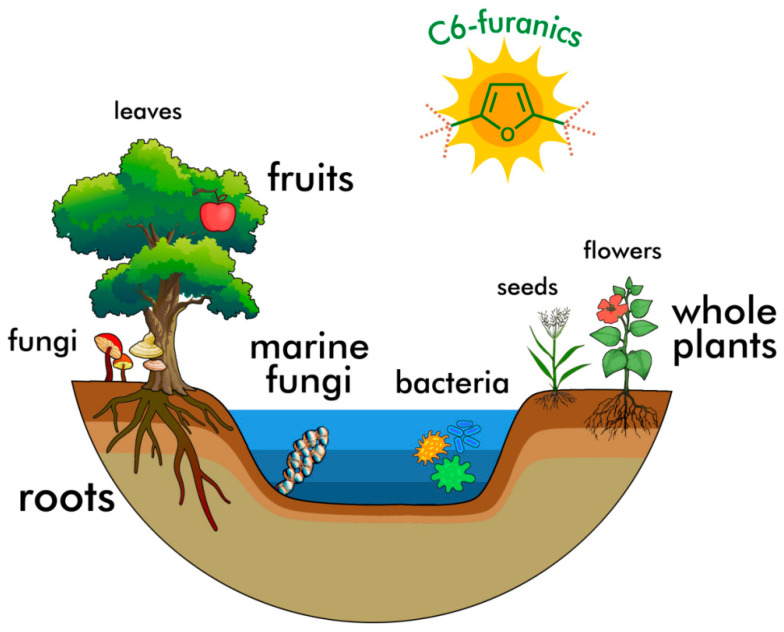
Schematic representation of the occurrence of C6-furanics in nature (font size corresponds to the prevalence of furanic derivatives in natural sources).

**Figure 3:**
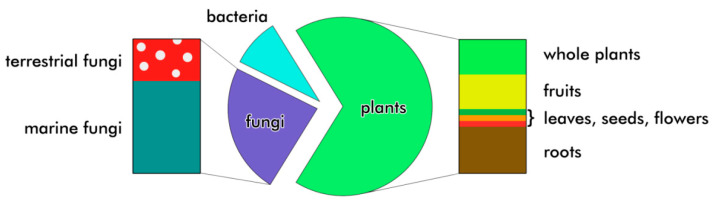
Relative distribution of C6-furan compounds in the biosphere: chart composition based on the number of isolated C6-furanics described in the literature.

Exploring the occurrence of C6-furanics in nature, several features come into consideration. Predominantly, such derivatives could be found in different parts of plants (Figure 2 and Figure 3), especially in the roots, rhizomes and fruits. It is logical considering that furan derivatives metabolically originate from carbohydrates, while roots and fruits are parts of plants that are richest in metabolically active carbohydrates. Additionally, several examples of furanic compounds isolated from fungi have been described, as well as a few bacteria-derived furans.

Since 5-HMF is a main product of the dehydration of hexoses, it is not surprising that HMF derivatives (esters, ethers, acetals, etc.) are the most common type of C6-furanics in nature. While HMF derivatives are predominantly located in plants, more oxidized as well as more reduced C6-furans are usually associated with fungi and bacteria. Therefore, it can be concluded that the chemical type of produced furanic metabolites strongly depends on the natural source.

The examples of naturally occurring C6-furanic compounds highlight a number of possible new opportunities. We believe that this review will inspire synthetic work on the preparation of biofurans and biological studies, which will establish the biological role of biofurans since, for many furanic compounds, it remains unclear.

## Data Availability

No new experimental data were created.
